# Case report: Interstitial lung disease of XELOEX chemotherapy with cetuximab in advanced colon cancer induced

**DOI:** 10.1097/MD.0000000000036379

**Published:** 2023-12-15

**Authors:** Yanfei Shao, Jieru Hu, Haibo Yao, Menglao Jiang, Zhouye Song

**Affiliations:** a Department of Pharmacy, Zhejiang Provincial People’s Hospital, Hangzhou, China; b Department of Pharmacy, Lishui Central Hospital and Fifth Affiliated Hospital of Wenzhou Medical College, Lishui, China; c Gastrointestinal Surgery, Zhejiang Provincial People’s Hospital, Hangzhou, China; d Zhejiang Center of Drug and Cosmetics Evaluation, Hangzhou, China; e Department of Pharmacy, Zhejiang Hospital, Hangzhou, China.

**Keywords:** cetuximab, China, colonic neoplasms, drug-induced abnormalities, interstitial, lung disease

## Abstract

**Introduction:**

This paper presents a case of a Chinese patient with advanced colon cancer who developed drug-induced interstitial lung disease while undergoing treatment with cetuximab combined with XELOX.

**Patient concerns:**

A 75-year-old man with a history of colon cancer, had metastases in the liver, peritoneum, and lungs, which were initially treated with XELOX and cetuximab (0.4 g) in 2019. However, the lung metastases progressed, and the cetuximab dosage was adjusted to 0.9 g and then readjusted to 0.4 g.

**Diagnosis:**

In January 2021, computed tomography revealed developed interstitial lung disease, leading to the discontinuation of chemotherapy and cetuximab.

**Interventions:**

Receiving methylprednisolone pulse therapy.

**Outcomes:**

The patient experienced respiratory failure and passed away. The Naranjo Algorithm Assessment score indicated a probable relationship between cetuximab and the adverse event.

**Conclusion:**

This case highlights the need for regular pulmonary imaging examinations during cetuximab therapy, as drug-induced interstitial lung disease may be associated with the dose and duration of treatment.

## 1. Introduction

Cetuximab is a chimeric monoclonal IgG1 antibody against the epithelial growth factor receptor, inhibiting tumor cell growth and inducing apoptosis. Cetuximab can be combined with the FOLFOX, FOLFIRI, and XELOX regimens.^[1]^ The recommended dose for RAS wild-type metastatic colorectal cancer treatment is 400 mg/m^2^ for the first dose, followed by 250 mg/m^2^ per week, 500 mg/m^2^ biweekly regimens recently appeared to be as effective as 1-week regimens.^[2]^ A particularly serious adverse reaction to cetuximab is a drug-induced interstitial lung disease (DILD).^[3,4]^ DILD-induced mortality is high due to irreversible disease progression and lack of effective treatment. There may be ethnic differences in the occurrence of DILD, with Japan reporting higher numbers than other countries.^[5]^ This paper presents a case of a DILD in a Chinese patient with advanced colon cancer treated with cetuximab combined with XELOX.

## 2. Case description

A 75-year-old man was admitted on January 11, 2021, after displaying cold symptoms for 3 days, including chest tightness, shortness of breath, low-grade fever, and mild dry cough. Oral compound aminophenolamine for 2 days did not relieve the symptoms.

The patient was diagnosed with colon cancer in 2015. In July 2019, computed tomography (CT) revealed multiple liver, peritoneum, and lung metastases. Molecular detection revealed that wild-type KRAS. As surgery was not an option (due to multiple metastases), an intravenous infusion port was implanted, and the patient received the XELOX regimen (21-day cycles; oxaliplatin 150 mg, d1; capecitabine 1.5 g, twice every day) combined with cetuximab (0.4 g, d1-8-15). The patient continued to smoke (approximately 40 cigarettes/day) and consume alcohol (700 mL/d) during chemotherapy. After 3 months of XELOX and cetuximab, CT reexamination revealed shrinkage of the liver and retroperitoneal metastases. The patient had a mild acne-like rash during treatment but reported no other discomfort.

From January to March 2020, the treatment was interrupted owing to COVID-19. In March 2020, CT revealed enlarged lung metastases and pulmonary interstitial fibrosis. The metastases shrunk again after restarting the XELOX chemotherapy. In September 2020, CT revealed that the lung metastases had increased, and cetuximab was adjusted to 0.9 g d1 (Table 1). The patient’s rash worsened, and CT showed no changes in the metastases.

**Table 1:** Changes in treatment and imaging.

Time	Chemotherapy regimen	Cetuximab	Changes in imaging
July 2019 to January 2020	XELOX (oxaliplatin 150 mg, once every 3 wk; capecitabine 1.5 g, twice every day)	0.4 g once a week	Slight fibrosis in both lungs
March 2020 to August 2020			Interstitial changes in both lungs (mainly fibrous foci)
September 2020 to November 2020		0.9 g once every 3 wk	Interstitial inflammatory changes in both lungs (mainly fibrous foci)
November 2020 to December 2020		0.4 g once a week	Interstitial inflammatory changes in both lungs (mainly fibrous foci), small patches of darkness
January 2021	Stop	Stop	Acute exacerbation of interstitial pneumonia

**Figure 1.**
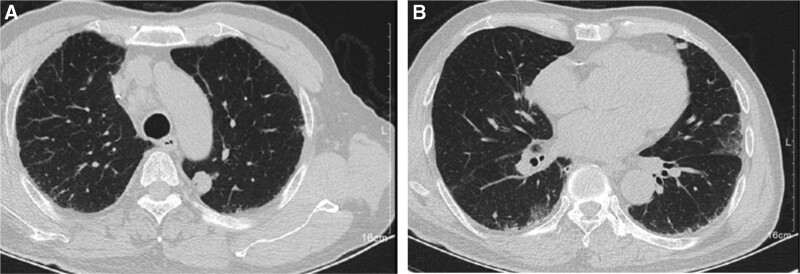
Chest computed tomography (CT) on November 28, 2020. Some ground-glass changes occurred in the lungs.

**Figure 2.**
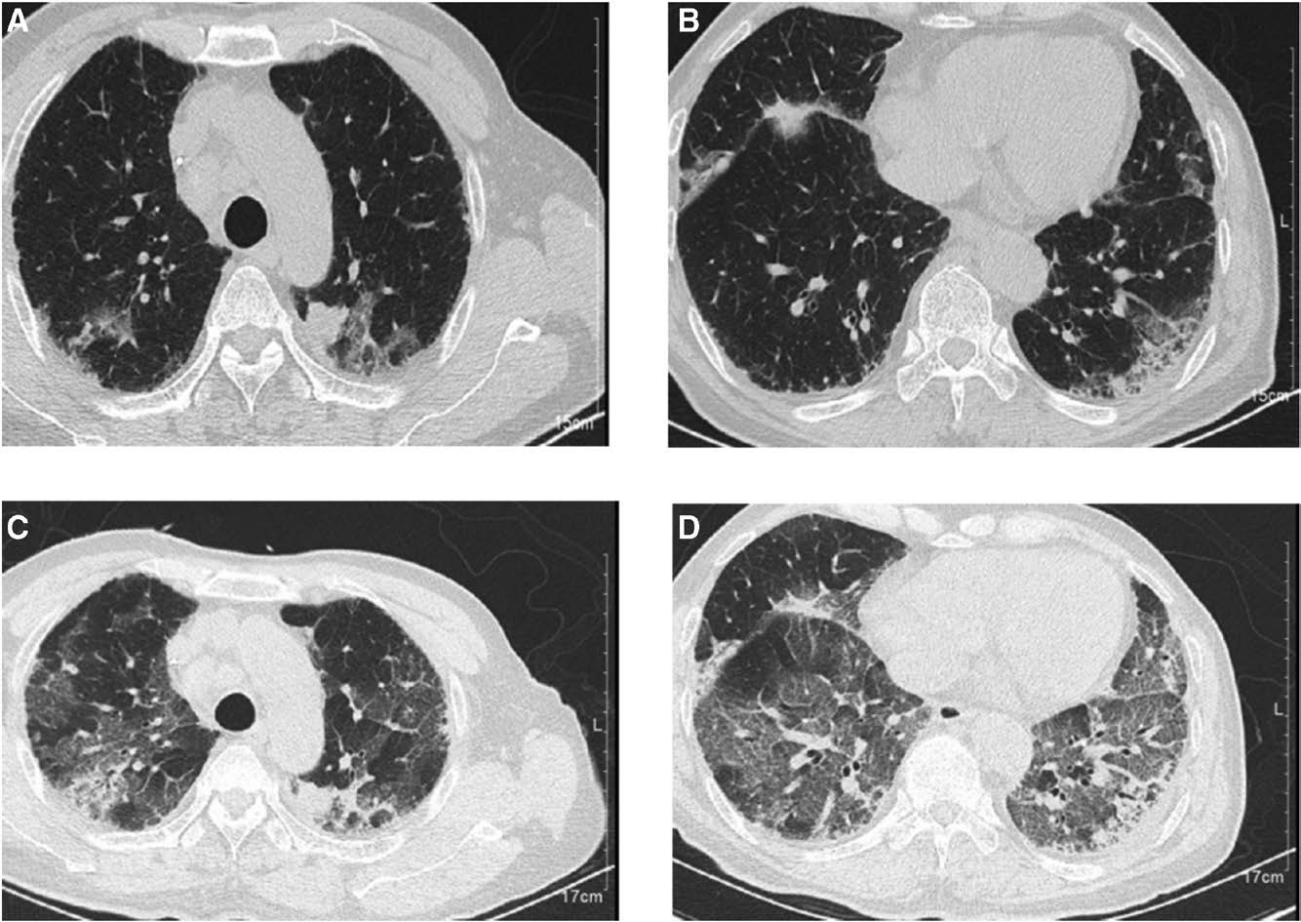
Chest computed tomography (CT) (A and B) January 6, 2021. (A) The 2 lungs were scattered in nodules, and the largest was located in the upper lobe of the left lung, with a size of 19 × 17 mm (increased metastasis). (B) The 2 lungs had distributed ground-glass flake shadows with local saw mesh-like cable strip high-density shadows, indicating local interstitial pneumonia (mainly fibrous lesions). (C and D) January 12, 2021. (C) The tumor size was the same, but the ground-glass flake shadows were increased in the 2 lungs. (D) Interstitial pneumonia in the 2 lungs had progressed, with pleural thickening, a small amount of pleural effusion, and atriotasi.

In November 2020, CT revealed small patches of darkness in the lungs (Fig. 1). As the tumors did not continue to grow, it was assumed that the changes in the lungs might be side effects of the drug. Therefore, cetuximab was adjusted to the original dose (0.4 g d1-8-15, Table 1). No clinical symptoms were observed after dose adjustment, but the pulmonary imaging did not improve. On January 6, 2021, CT revealed that the 2 lungs had distributed ground-glass flake shadows and local saw mesh-like cable strip high-density shadows, indicating local interstitial lung disease (ILD) (mainly fibrous lesions) (Fig. 2A and B). Chemotherapy and cetuximab were discontinued (Table 1).

Respiratory failure occurred on January 12, 2021, and he was transferred to the intensive care unit (ICU). CT revealed that the metastases remained the same, but the ground-glass flake shadows were increased in the 2 lungs, with interstitial pneumonia progression (Fig. 2C and D). In the ICU, the patient was given high-flow respiratory support and endotracheal intubation with mechanical ventilation. He was kept on empirical anti-infection and anti-pneumocystis pneumonia treatment, but the condition did not improve. Tests for Aspergillus, Mycobacterium, Cryptococcus, Candida, or other common respiratory tract drug-resistant bacteria or fungi were negative. After a multidisciplinary discussion, the doctors ruled out infectious pneumonia and immune-related connective tissue disease. The fibrotic lesions continued to progress and failed to respond to treatments. Methylprednisolone (500 mg/d) pulse therapy was administered for 3 days; the patient improved slightly for a short time but eventually died on January 20, 2021.

## 3. Discussion

According to the WHO’s definition of the causality of adverse reactions, the ILD adverse reaction could have been caused by either cetuximab or oxaliplatin. However, by investigating the relationship between the use of the 2 drugs and the changes in lung imaging, the pharmacist concluded that cetuximab was likely to be the main cause of the ILD in this case. First, cetuximab-induced ILD is more common (0.1–1%) than oxaliplatin-induced ILD (0.01–0.1%). Second, DILD occurred after cetuximab was initiated and aggravated after increasing the cetuximab dose. Third, the rare occurrence of oxaliplatin-induced ILD is predominantly acute and generally not inducible. In the case reported here, ILD seemed to change from chronic to acute exacerbation triggered by a common cold, as previously observed.^[5]^ Of note, the patient was negative for COVID-19 (nucleic acid test), which was a mandatory test in 2021 after hospital admission. Finally, the Naranjo Algorithm Assessment score was 6, meaning that the probability that the adverse event was related to cetuximab was classified as probable (Table S1, Supplemental Digital Content, http://links.lww.com/MD/K923).

A post-marketing retrospective study in Japan revealed patients with ILD caused by cetuximab.^[6]^ Satoh et al^[7]^ reported that the incidence of ILD in Japanese patients with colorectal cancer treated with cetuximab was 1.2%, higher than in other countries (0.1–0.6%), suggesting possible ethnic differences in the incidence of cetuximab-induced DILD. In patients with head and neck cancer, the incidence of cetuximab-induced DILD might even be higher (4.5–9.5%).^[3,8]^ These studies also suggested likely risk factors related to cetuximab-induced DILD. Indeed, patients >65 years with a history of ILD had a significantly high recurrence rate.^[7]^ Other possible risk factors include lung metastases, high baseline KL-6, high smoking index, squamous-cell carcinoma, and ≥2 prior chemotherapy regimens (vs ≤1).^[3,7]^ The patient reported here had a low level of lung fibrosis before cetuximab, but no ILD was diagnosed, but he was > 65 years old, had lung metastases, and had a history of heavy smoking.

Clinical trials of the biweekly cetuximab regimen were conducted, and the FDA approved the 500 mg/m^2^ cetuximab biweekly regimen because the efficacy results were equivalent to that of a single weekly regimen.^[9,10]^ On the other hand, these clinical trials did not address the question of increased toxicity. In fact, the literature suggests that cetuximab accumulates when the dosage exceeds 400 mg/m^2^.^[11]^ In the case reported here, the adverse reactions were aggravated even when the dosage of 542 mg/m^2^ was used once every 3 weeks (the standard biweekly regimen was not approved at that time, and it was approved in April 2021, and the off-label use was done with the patient’s consent). The patient developed a severe rash with pulmonary fibrous foci. The DILD toxicity of cetuximab might be dose-dependent, and the biweekly regimen is now approved for use, but the concerns over the development of DILD should remain.^[3,5–8]^

Interstitial pneumonia and pulmonary fibrosis are the most common manifestations of DILD in a lung CT scan. The clinical manifestations of cetuximab-induced DILD are similar to those of general drug-induced lung injury, i.e., dyspnea (in approximately 90% of patients), cough, and fever.^[4]^ Therefore, the patient reported here displayed the typical clinical manifestations of ILD. Still, it has to be stressed that the imaging manifestations appear before the clinical manifestations and that when the clinical symptoms appear, it might be too late to start treatments. The mechanisms of cetuximab-induced DILD remain unclear,^[12]^ preventing effective treatments. DILD tends to progress rapidly and has a high mortality rate.^[7]^ Still, there are no standard recommendations for the treatment of DILD. General treatment includes stopping suspicious drug exposure and giving oxygen therapy, and data obtained from glucocorticoid treatment therapies are controversial. A better strategy would be to practice prevention methods, including pneumonia/flu vaccines, avoidance of triggers, identification of risk factors, and regular chest CT examinations.^[4]^

## 4. Conclusions

DILD caused by cetuximab can be severe, rapidly progressive, and fatal, and there is no suitable treatment. Therefore, the cetuximab dose cannot be arbitrarily increased when the treatment response is not satisfactory. Meanwhile, physicians should pay attention to changes in lung imaging because patients may not have obvious clinical symptoms in the early stages. Early identification of DILD can avoid tragic outcomes.

## Author contributions

**Writing – original draft:** Yanfei Shao, Jieru Hu, Haibo Yao, Menglao Jiang, Zhouye Song.

## Supplementary Material


