# Supplementary material for: Case report: Interstitial lung disease of XELOEX chemotherapy with cetuximab in advanced colon cancer induced
Source: Medicine (Baltimore). 2023 Dec 15;102(50):e36379. doi: 10.1097/MD.0000000000036379 (PMC10727633; doi:10.1097/MD.0000000000036379)
Supplement: Supplementary file 1 [file medi-102-e36379-s001.docx]

**Supplementary Table S1.** Cetuximab adverse drug reactions probability scale

|  | Yes | No | Do not know | Score | Reason |
| --- | --- | --- | --- | --- | --- |
| 1. Are there previous conclusive reports on this reaction? | 1 | 0 | 0 | 1 | There have been clinical reports of  cetuximab-induced ILD |
| 2. Did the adverse event appear after the suspected drug was administered? | 2 | -1 | 0 | 2 | ILD appeared after cetuximab therapy |
| 3. Did the adverse reaction improve when the drug was discontinued, or a specific antagonist was administered? | 1 | 0 | 0 | 0 | ILD did not improve |
| 4. Did the adverse reaction reappear when the drug was readministered? | 2 | -1 | 0 | 0 | Cetuximab therapy was not readministered |
| 5. Are there alternative causes (other than the drug) that could, on their own, have caused the reaction? | -1 | 2 | 0 | 2 | The doctor ruled out infection or immune causes |
| 6. Did the reaction reappear when a placebo was given? | -1 | 1 | 0 | 0 | Placebo was not given |
| 7. Was the drug detected in the blood (or other fluids) in concentrations known to be toxic? | 1 | 0 | 0 | 0 | Cetuximab concentration was not detected |
| 8. Was the reaction more severe when the dose was increased or less severe when the dose was decreased? | 1 | 0 | 0 | 1 | The reaction was more severe when the dose of cetuximab was increased. |
| 9. Did the patient have a similar reaction to the same or similar drugs in any previous exposure? | 1 | 0 | 0 | 0 | The patient has not declared a similar reaction before. |
| 10. Was the adverse event confirmed by any objective evidence? | 1 | 0 | 0 | 0 | No |
|  |  |  |  | Total: 6 |  |
